# Structural basis for the binding of the cancer targeting scorpion toxin, ClTx, to the vascular endothelia growth factor receptor neuropilin-1

**DOI:** 10.1016/j.crstbi.2021.07.003

**Published:** 2021-08-02

**Authors:** Gagan Sharma, Carolyne B. Braga, Kai-En Chen, Xinying Jia, Venkatraman Ramanujam, Brett M. Collins, Roberto Rittner, Mehdi Mobli

**Affiliations:** a Centre for Advanced Imaging, The University of Queensland, St Lucia, QLD, Australia; b Chemistry Institute, University of Campinas, P.O. Box 6154, 13083-970, Campinas, SP, Brazil; c Institute for Molecular Bioscience, The University of Queensland, St Lucia, QLD, Australia

**Keywords:** Chlorotoxin, NRP1, Disulfide-rich peptide, Heteronuclear NMR, ITC

## Abstract

Chlorotoxin (ClTx) is a 36-residue disulfide-rich peptide isolated from the venom of the scorpion *Leiurus quinquestriatus.* This peptide has been shown to selectively bind to brain tumours (gliomas), however, with conflicting reports regarding its direct cellular target. Recently, the vascular endothelial growth factor receptor, neuropilin-1 (NRP1) has emerged as a potential target of the peptide. Here, we sought to characterize the details of the binding of ClTx to the b1-domain of NRP1 (NRP1-b1) using solution state nuclear magnetic resonance (NMR) spectroscopy. The 3D structure of the isotope labelled peptide was solved using multidimensional heteronuclear NMR spectroscopy to produce a well-resolved structural ensemble. The structure points to three putative protein-protein interaction interfaces, two basic patches (R14/K15/K23 and R25/K27/R36) and a hydrophobic patch (F6/T7/T8/H10). The NRP1-b1 binding interface of ClTx was elucidated using ^15^N chemical shift mapping and included the R25/K27/R36 region of the peptide. The thermodynamics of binding was determined using isothermal titration calorimetry (ITC). In both NMR and ITC measurements, the binding was shown to be competitive with a known NRP1-b1 inhibitor. Finally, combining all of this data we generate a model of the ClTx:NRP1-b1 complex. The data shows that the peptide binds to the same region of NRP1 that is used by the SARS-CoV-2 virus for cell entry, however, via a non-canonical binding mode. Our results provide evidence for a non-standard NRP1 binding motif, while also providing a basis for further engineering of ClTx to generate peptides with improved NRP1 binding for future biomedical applications.

## Highlights

• Structural details of the binding of the scorpion toxin, chlorotoxin (ClTx) to the VEGF receptor neuropilin-1 (NRP1).
• ClTx was produced in its native fold and isotope labelled using a new, high-yield, bacterial expression system.
• Multidimensional heteronuclear NMR experiments reveal the high-resolution structure of ClTx and its binding to NRP1.
• ClTx binds to NRP1 via a non-canonical primary sequence that satisfies the receptor binding motif through its tertiary fold.

## Graphical abstract

**Figure:**
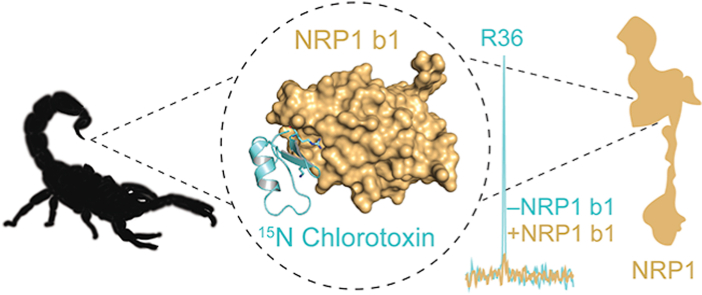

## 1 Introduction

Disulfide-rich peptides found in the venom of animals including snakes, spiders, scorpions and cone snails have been increasingly considered as promising templates for novel therapeutic drug development (Harvey, 2014). This is due to the fact that, in addition to their potent and specific interactions with a wide variety of physiological targets, they have enhanced stability and structural complexity compared to linear peptides (Gongora-Benitez et al., 2014). These venom peptides have also been extensively used to characterize receptor function and pharmacology, shaping our view of many physiological processes (Vetter et al., 2016).

Scorpion venoms are a well-known source of disulfide-rich toxins and have been extensively explored for discovery of drug candidates for the treatment of cancers, mainly after the discovery of chlorotoxin (ClTx) in the early 1990s. ClTx is a bioactive 36-amino acid residue peptide stabilized by four disulfide bonds, isolated from the venom of a scorpion, *Leiurus quinquestriatus* (DeBin and Strichartz, 1991; DeBin et al., 1993), which has been demonstrated to preferentially target cancerous tissues, including malignant brain tumours – gliomas (Lyons et al., 2002).

The extraordinary selectivity shown by this peptide towards cancer cells, and in particular to common brain tumours (glioblastoma - GBM), has attracted extensive interest for its use in cancer diagnosis and therapy. A synthetic ClTx labelled with ^131^I (commercial name ^131^I-TM-601) has already undergone early phase clinical trials and has received FDA approval for a phase III trial in patients with newly diagnosed gliomas (Jacoby et al., 2010; Mamelak and Jacoby, 2007; Mamelak et al., 2006). In addition, other ClTx-based conjugates and nanoparticles have been developed for tumour imaging and targeting (Fu et al., 2012; Stroud et al., 2011; Veiseh et al., 2005, 2007; Zhao et al., 2015). Most recently a chimeric antigen receptor (CAR) T cell therapy was devised using ClTx for cancer targeting, showing excellent selectivity and regression in tumour xenograft models of GBM (Wang et al., 2020).

The mechanism by which ClTx targets gliomas remains under some debate. Several candidates have been identified and claimed to be the primary cell surface target. Pharmacological characterisation of ClTx initially revealed it to be a potent blocker of small conductance epithelial chloride channels, from which its name was derived (DeBin et al., 1993). Still, in this initial report, the authors observed an inhibition only when the molecule was applied to the intracellular surface of these ion channels. They, thus, concluded that it would appear unlikely that chloride channels under normal conditions would be the molecular target of the toxin. Subsequently additional cell surface proteins were suggested to be target of ClTx including a glioma-specific chloride channel (GCC) (Ullrich et al., 1996, 1998; Ullrich and Sontheimer, 1996), matrix metalloprotease-2 receptor (MMP-2) (Cox and O'Byrne, 2001; Deshane et al., 2003; Sternlicht and Werb, 2001) and annexin A2 (Kesavan et al., 2010).

Most recently, the vascular endothelial growth factor (VEGF) receptor, Neuropilin-1 (NRP1) was identified as the likely receptor, responsible for tumour uptake (McGonigle et al., 2019). The extracellular b1 domain of NRP1 (NRP1-b1) is known to bind to VEGF via a C-terminal basic region including a terminal arginine residue. Detailed analysis of the binding of peptides to NRP1 revealed a consensus sequence, leading to the definition of the C-end rule, which states that a C-terminal sequence [R or K] XX [R or K], where X is any amino acid, is recognised by NRP1 for internalisation (Teesalu et al., 2009). NRP1 is overexpressed in many cancers but naturally upregulated in lung and heart tissue (Rossignol et al., 2000). Indeed, many pathogens are known to use this consensus sequence to hijack this natural internalisation process to enter into mammalian cells. Notably, it has been shown that cleavage of the spike protein of SARS-CoV-2 by furin exposes a peptide that binds directly to the NRP1-b1 domain and that blocking this interaction results in reduced infection of cell cultures (Cantuti-Castelvetri et al., 2020; Daly et al., 2020).

Although ClTx does not contain the [R/K]XX[R/K] motif it was shown to bind the NRP1-b1 domain, but only when the peptide was deamidated to expose the carboxy group of its C-terminal arginine residue (McGonigle et al., 2019). This raises the question of how ClTx can bind to NRP1, despite not adhering to the C-end rule. To address this question, we have used a combination of multidimensional, heteronuclear NMR spectroscopy and isothermal titration calorimetry (ITC) to investigate the structural and thermodynamic details of the binding of ClTx to NRP1. Combining this data with competition data using a known inhibitor of NRP1, we define the binding interface of both molecules and use this information to generate a docking model of the complex. Our data indicates an unusual binding mode, which suggests that while the [R/K]XX[R/K] motif is not satisfied in the primary sequence of ClTx, the 3D arrangement of basic residues in the peptide indirectly fulfils this requirement, indicating a new cryptic NRP1-binding mode in highly constrained globular peptides such as ClTx.

## 2 Results

### 2.1 Chlorotoxin production

**Fig. 1:**
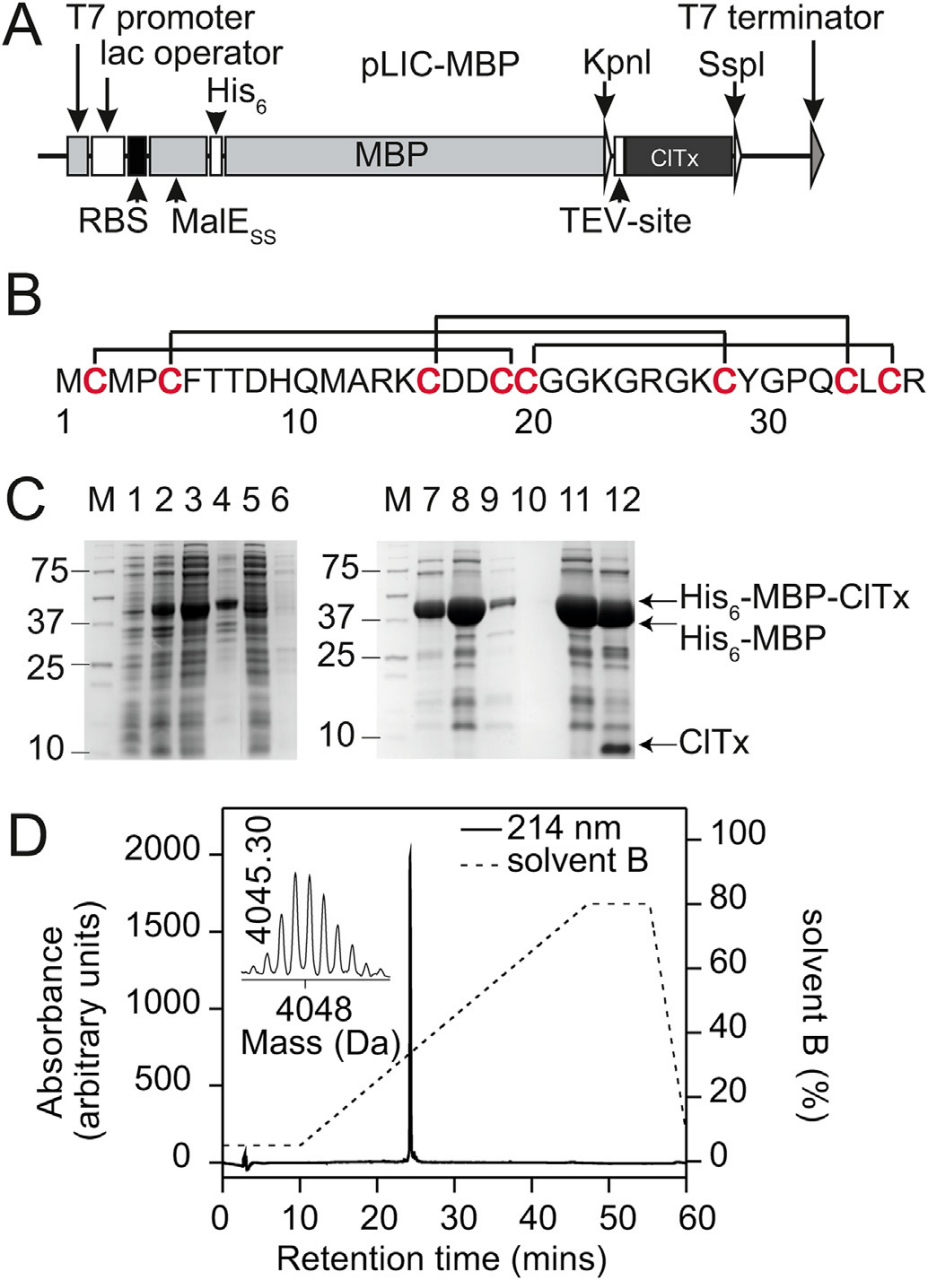
**Recombinant production of ClTx. A** Schematic representation of the pLIC-MBP vector used for periplasmic expression of ClTx. The coding region includes a MalE signal sequence (MalE_SS_) for periplasmic export, a His_6_ affinity tag, a maltose binding protein (MBP) fusion tag and a codon-optimized gene encoding ClTx, with a TEV protease recognition site inserted between the MBP and toxin-coding regions. The locations of key elements of the vector are shown, including the ribosome-binding site (RBS), T7 promoter and lac operator. **B)** Primary structure of ClTx. Disulfide bridge connectivities are shown above the sequence. **C)** Coomassie stained SDS-PAGE gels analysis illustrating different steps in the expression and purification of ClTx. Lanes contain: M, molecular weight markers (the masses of the selected standards in kDa is shown on the left); lane 1, *E. coli* cell extract prior IPTG induction; lane 2, *E. coli* cell extract after IPTG induction; lane 3, soluble cell extract after sonication; lane 4, insoluble fraction after sonication; lane 5, flow through after loading the cell lysate into Ni-NTA column; lane 6, eluate from washing Ni-NTA column with TN buffer; lane 7, 1st eluate from washing Ni-NTA column with 250 ​mM imidazole buffer; lane 8, 2nd eluate from washing Ni-NTA column with 250 ​mM imidazole buffer; lane 9, 3rd eluate from washing Ni-NTA column with 250 ​mM imidazole buffer; lane 10, eluate from washing Ni-NTA column with 1 ​M imidazole; lane 11, concentrated and desalted fusion protein sample before TEV protease cleavage; lane 12, sample after TEV protease cleavage showing almost complete cleavage of the His_6_-MBP-ClTx fusion protein. **D)** RP-HPLC chromatogram showing the final step in the purification of correctly folded recombinant ClTx. The dotted line (---) indicates the gradient of the solvent B. Elution of protein was monitored at 214 ​nm. Inset is a MALDI-TOF MS spectrum showing the [M+H]^+^ ion for the purified ^15^N-labelled recombinant toxin (monoisotopic mass measured 4045.3 agrees well with the calculated value of 4048.75).

Disulfide-rich peptides pose a particular problem in recombinant expression. The highly reducing environment of the cytoplasm prevents formation of disulfide bonds resulting in either poor yields or misfolded peptides. An approach that has proved successful for bacterial expression of disulfide-rich spider toxins is to incorporate a signal sequence that sends the nascent peptide to the periplasm, where the enzymes involved in disulfide-bond formation and shuffling are located (Klint et al., 2013). Thus, to produce ClTx, we employed an isopropyl-β-D-thiogalactopyranoside (IPTG)-inducible construct (Fig. 1A) that allowed export of a His_6_-MBP-toxin fusion protein to the *E. coli* periplasm. Using this expression system, a significant amount of His_6_-MBP-toxin fusion protein was recovered in the soluble cell fraction following IPTG induction.

The His_6_-MBP-ClTx fusion protein was purified using nickel affinity chromatography (Fig. 1C, lanes 1–10). There was minimal loss of fusion protein in the flow-through from nickel beads (Fig. 1C, lanes 5–6). The bound fusion protein was eluted from the column and cleaved with a His_6_-tagged TEV protease (Fig. 1C, lanes 7–8). The His_6_-tagged MBP and TEV protease were then removed by passage through a Ni-NTA column, and ClTx subsequently purified using reverse-phase (RP) HPLC. ClTx eluted as a single, major, fully oxidized isomer with a retention time of ~25 ​min under the described experimental conditions. The purity of ClTx was assessed to be >98% as judged by RP-HPLC and matrix-assisted laser-desorption ionization time-of-flight mass spectrometry (MALDI-TOF MS) (Fig. 1D, inset). The final yield of ClTx was ~2 ​mg of toxin per litre of culture. This yield is similar to that previously reported for a GST tagged version of the peptide. However, in the previous report, the fusion protein was purified from the cytoplasm and required an additional refolding step (Wang et al., 2013).

### 2.2 High-resolution solution structure of ClTx

**Fig. 2:**
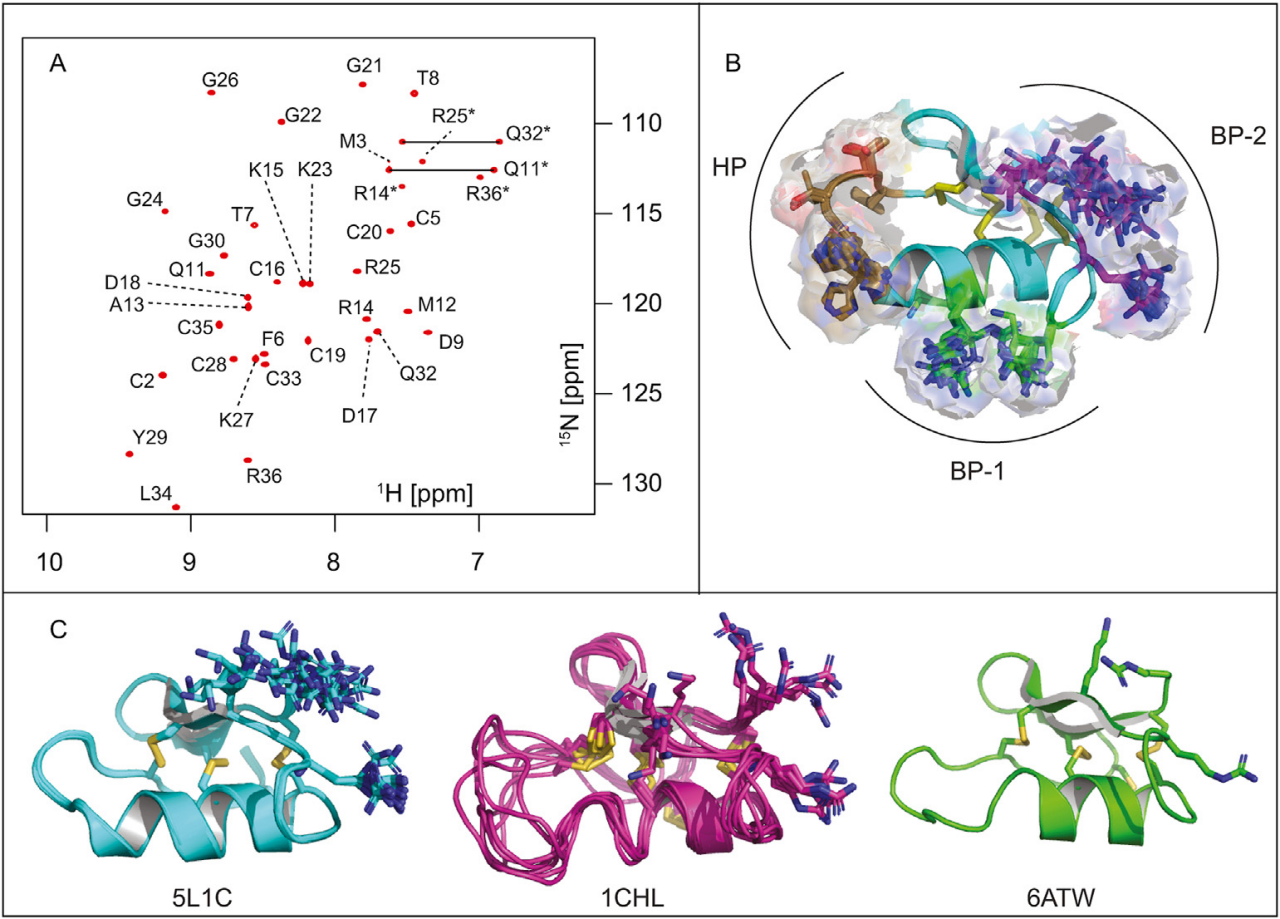
^**15**^**N-HSQC and 3D structure of ClTx. A)** Assigned ^1^H–^15^N HSQC of ClTx where sidechain NHs are indicated with an asterisk (∗). **B)** Putative bioactive surfaces of ClTx. The peptide contains three distinct surfaces, two of these are basic patches (BP-1 in green and BP-2 magenta), rich in lysines and arginines, whilst the third is a weakly hydrophobic patch (HPFig. 5, shown in brown). All three surfaces are on the same side of the peptide while the opposite side contains the cysteine scaffold. **C)** Side-by-side comparison of the heteronuclear NMR structure (5L1C) reported here, with the homonuclear structure (1CHL), and crystal structure (6ATW). The disulfide bonds are indicated with yellow sticks. The BP-2 sidechains are shown in sticks, revealing a different orientation of K27 in 1CHL and thus a change in this basic patch. (For interpretation of the references to colour in this figure legend, the reader is referred to the Web version of this article.)

NMR data were collected at 25 ​°C using ^13^C/^15^N-labelled ClTx dissolved in 20 ​mM phosphate buffer, pH 5.8. ^1^H_N_, ^15^N, ^13^C_α_, ^13^C_β_, and ^13^C’ resonance assignments were obtained from analysis of amide-proton strips in 3D HNCACB, CBCA(CO)NH, and HNCO spectra. Sidechain ^1^H and ^13^C chemical shifts were obtained using a 4D HCC(CO)NH-TOCSY experiment, simultaneously providing sidechain ^1^H–^13^C connectivities (Mobli et al., 2010). A fully assigned ^1^H–^15^N-HSQC spectrum of ClTx is shown in Fig. 2. Complete chemical shift assignments have been deposited in BioMagResBank (Accession Number 30149).

CYANA was used for automated NOESY assignment and structure calculation (Güntert, 2004). Disulfide-bond connectivities were determined from ^15^N- and ^13^C-NOESY spectra and confirmed by performing structure calculations without inclusion of disulfide-bond restraints (Mobli and King, 2010). The following connectivities were unambiguously established following this procedure: Cys2-Cys19, Cys5-Cys28, Cys16-Cys33, and Cys20-Cys35, which corresponds to a 1–4, 2–6, 3–7, 5–8 framework, as previously described (Lippens et al., 1995). For structure calculations, interproton distance restraints were supplemented with 57 dihedral-angle restraints (28 φ, 29 ψ) derived from TALOS ​+ ​chemical shift analysis (Shen et al., 2009). 200 structures were calculated from random starting conformations and then the 20 structures with the lowest target function values were selected to represent the solution structure of ClTx. Coordinates for the final ensemble of structures have been deposited in the Protein Data Bank (Accession Number 5L1C). The 20 structures overlay very well, with backbone and heavy-atom RMSDs of 0.12 ​Å and 0.70 ​Å, respectively. Based on precision and Ramachandran plot quality (see Table 1), the structure ranks as high resolution (Kwan et al., 2011). The structure has high stereochemical quality with a mean MolProbity (Chen et al., 2010) score of 1.89 ​± ​0.19, placing it in the 80^th^ percentile relative to other protein structures.

**Table 1:** NMR and refinement statistics for 20 structures of chlorotoxin. The stereochemical quality is reported according to MolProbity (http://helix.research.duhs.duke.edu). The clash score is the number of steric overlaps >0.4 ​Å per 10^3^ atoms. All statistics are given as mean ​± ​standard deviation.

PDB ID	5L1C
BMRB ID	30149
**Experimental restraints**	
Inter-proton distance restraints	
Total	533
Intra-residue (*i ​= ​j*)	147
Sequential (|*i - j|* ​= ​1)	154
Medium range (1 ​< ​|*i – j|* ​< ​5)	134
Long range (|*i – j|* ​≥ ​5)	98
Disulfide bond restraints	12
Dihedral-angle restraints (φ, ψ)	57 (28,29)
Total number of restraints per residue	16.7
**RMSD to mean coordinate structure, Å**	
All Backbone atoms	0.12 ​± ​0.04
All heavy atoms	0.70 ​± ​0.11
Backbone atoms (Residues 2–5,9–35)	0.07 ​± ​0.03
Heavy atoms (Residues 2–5,9–35)	0.64 ​± ​0.10
**Stereochemical quality**	
Ramachandran plot statistics	
Most favored region, %	96.9 ​± ​0.66
Disallowed regions, %	0 ​± ​0
Unfavorable sidechain rotamers, %	8.33 ​± ​4.65
Clashscore, all atoms	2.89 ​± ​1.32
Overall MolProbity score (percentile)	80.6 ​± ​8.57

The alignment of the new structure (5L1C) with the existing structures (1CHL & 6ATW) shows good general agreement, with an RMSD of 1.5 ​Å and 0.9 ​Å over the backbone atoms. The discrepancy to the older NMR structure stems largely from disorder in a loop of 1CHL spanning residues 6–11 (Fig. 2C). As expected, the new structure based on a larger number of structural restraints derived from less ambiguous heteroatom edited NOESY spectra and chemical shift based dihedral angle predictions is better defined overall and agrees very well with the reported crystal structure (Fig. 2C) (Correnti et al., 2018).

### 2.3 NMR chemical shift mapping of ClTx – Neuropilin interactions and competition with EG00229

**Fig. 3:**
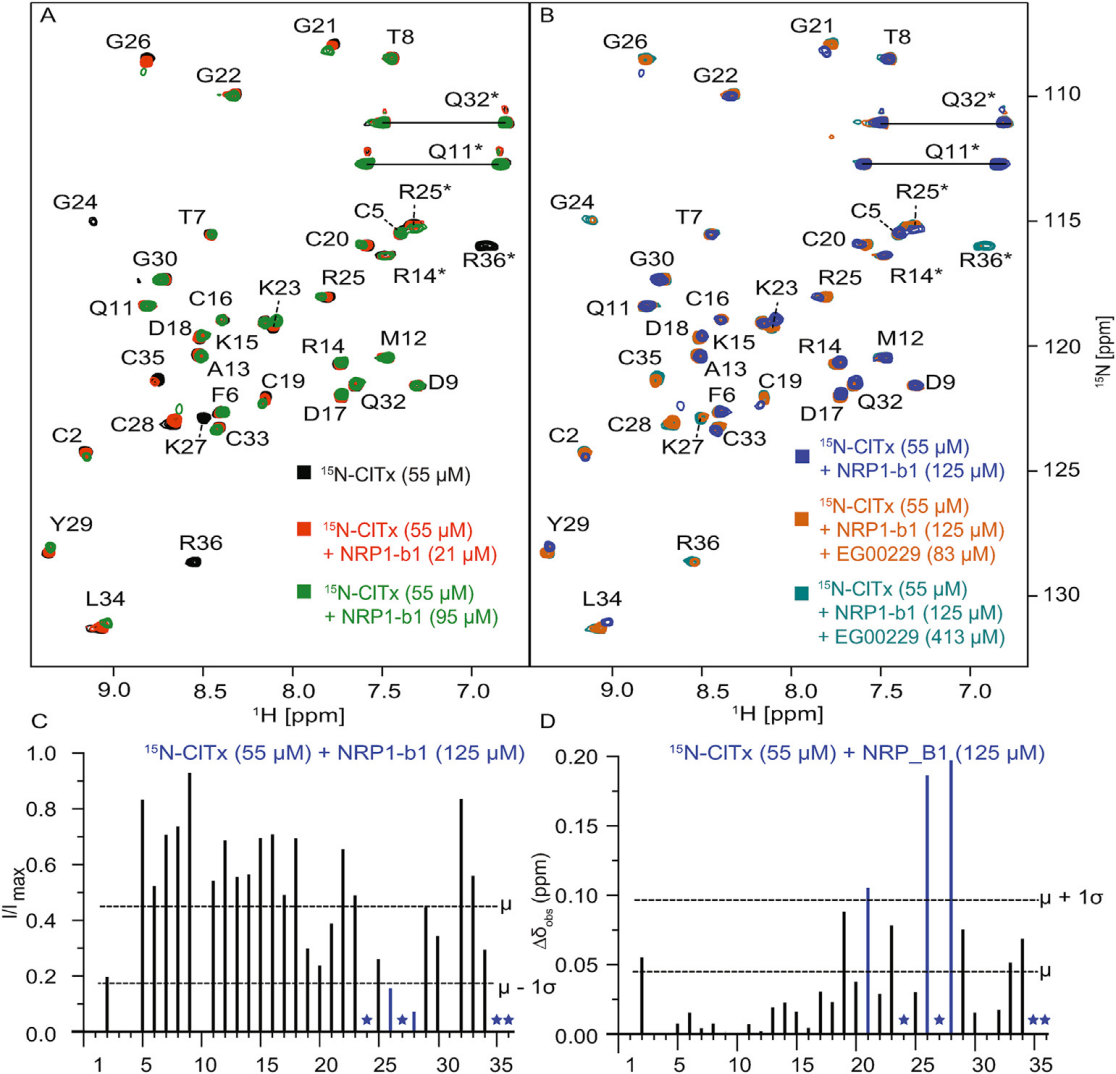
**NMR chemical shift mapping experiments. A)**^1^H–^15^N HSQC spectra of ClTx at a concentration of 55 ​μM in the NMR buffer (50 ​mM sodium citrate pH 5.5, 150 ​mM NaCl, 5% D_2_O). Reference spectrum (without addition of NRP1-b1) is shown in black and is superimposed with spectra upon addition of NRP1-b1 at two concentrations (orange, 21 ​μM; green, 95 ​μM). **B)**^1^H–^15^N HSQC spectra of ClTx at a concentration of 55 ​μM in the NMR buffer (50 ​mM sodium citrate pH 5.5, 150 ​mM NaCl, 5% D_2_O) containing NRP1-b1 at the concentration of 125 ​μM. Reference spectrum (without addition of EG00229) is shown in blue and is superimposed with spectra upon addition of EG00229 at two concentrations (brown, 83 ​μM; teal, 413 ​μM). **C)** Quantitation of resonance intensity changes, where dotted lines indicate the average change (μ) and one standard deviation (σ) subtracted from this change. **D)** Quantitation of chemical shift changes, where dotted lines indicate the average change (μ) and one standard deviation (σ) above this change. (For interpretation of the references to colour in this figure legend, the reader is referred to the Web version of this article.)

Chemical shift perturbation experiments were performed by increasing additions of NRP1-b1 to a fixed concentration of ^15^N-labelled ClTx. During these titrations, changes in peak intensity or chemical shifts were observed when compared to the ^15^N spectrum of ClTx in absence of NRP1-b1. The [^15^N-ClTx]-[NRP1-b1] titrations led to changes in chemical shifts of amino acids: 2C, 20C, 21G, 23K, 25R, 26G, 28C, 29Y and 34L (Fig. 3). Similarly, changes in peak intensity were observed for the amino acids – 2C, 25R, 26G, 27K, 28C, 35C, and 36R (Fig. 3). Upon addition of 413 ​μM of a small molecule inhibitor of NRP1-b1 (EG00229) (Jarvis et al., 2010), backbone signal of ^15^N ClTx were completely recovered, indicating overlapping NRP1-b1 binding interfaces shared between ClTx and EG00229 (Fig. 3).

### 2.4 ITC measurements of ClTx – Neuropilin-1 interactions and competition with EG00229

**Fig. 4:**
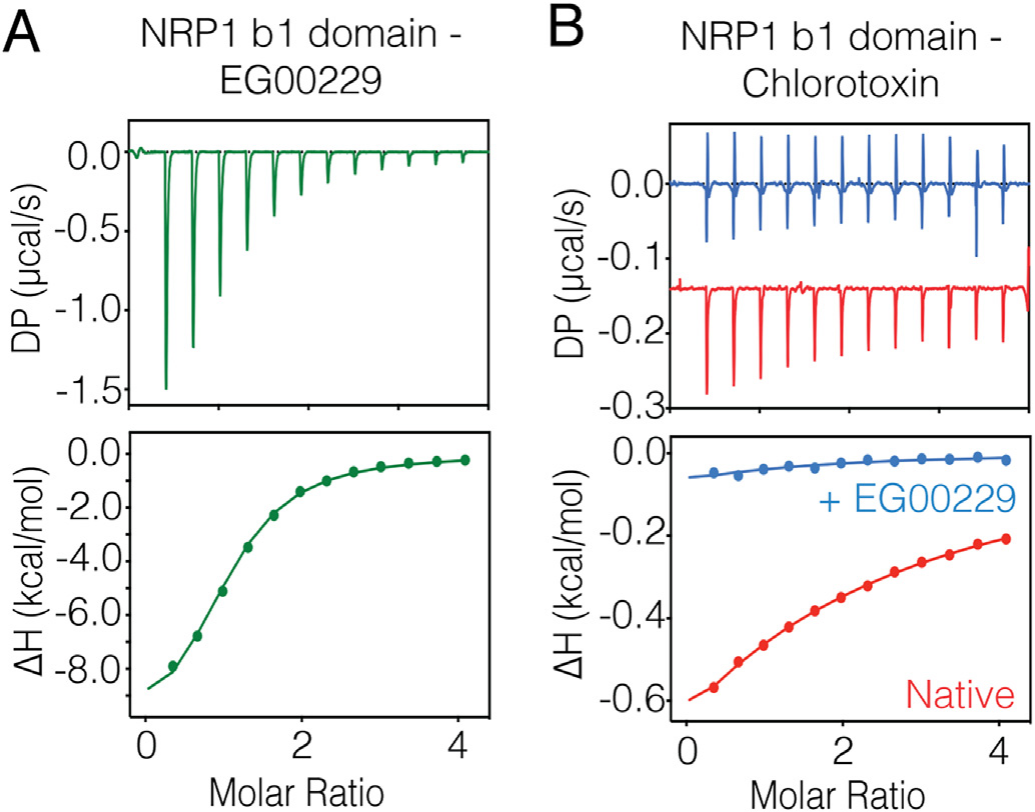
**Chlorotoxin binds weakly to the b1 domain of NRP1. A)** ITC thermogram showing the strong binding of EG00229 inhibitor to the b1 domain of NPR1. **B)** Chlorotoxin binds to the b1 domain of NRP1 with much weaker affinity. Addition of EG00229 to the b1 domain of NRP1 completely blocks chlorotoxin from binding. All ITC graphs represent the integrated and normalized data fit with 1 to 1 ratio of binding.

ITC revealed that small molecule inhibitor EG00229 binds to NRP1-b1 with a *K*_d_ of 7.9 ​μM (Fig. 4A and Table 2), which is in agreement with a previous study (Jarvis et al., 2010). Under the same experimental conditions, we found that CITx binds to NRP1-b1 more weakly, with an affinity of 143 ​μM, and that the interaction is fully inhibited by addition of EG00229, suggesting that ClTx is binding to the same site as canonical C-end rule peptides (Fig. 4B and Table 2).

**Table 2:** Thermodynamic parameters for the binding of NRP1-b1 domain with chlorotoxin compares to the EG00229 inhibitor.

	*K*_d_ (μM)	Δ*H* (kcal/mol)	Δ*G* (kcal/mol)	*-*TΔ*S* (kcal/mol)	N
EG00229	7.9 ​± ​0.8	−10.0 ​± ​0.2	−7.1 ​± ​0.06	2.9 ​± ​0.2	1.0
Chlorotoxin	143.3 ​± ​3.2	−2.4 ​± ​0.2	−5.4 ​± ​0.02	−3.0 ​± ​0.2	1.0

### 2.5 Modelling of the ClTx – Neuropilin-1 b1 domain complex

The chemical shift mapping experiments reveal that residues R36 and K27 were most perturbed by addition of NRP1-b1 (excluding G/C residues). The perturbation of the G and C residues (G24, G26, C28, C35) further indicated that sections 24-28 and 35–36 may be affected by conformational exchange. These residues were, therefore, included as flexible segments in the docking. The competition data shows that the binding of ClTx and EG00229 on NRP1-b1 are overlapping. From this we assumed that the C-terminal arginine of ClTx would occupy the highly conserved NRP1-b1 arginine binding site. Initial docking using only ambiguous restraints between the two proteins (based on chemical shifts and known NRP1 complexes 5IJR/4DEC) yielded no clusters (of complexes) where ClTx occupied the highly conserved binding interface of NRP1-b1. In a subsequent round of docking, we introduced unambiguous restraints to reduce the available conformational sampling space by orienting the basic residues of ClTx with the known binding interface of NRP1-b1. This pose was fixed by introducing three unambiguous constraints between the two proteins. The first was placed between the carbonyl carbon of residue 36 in ClTx and the W301 indole nitrogen (NE1) of NRP1-b1, constrained between 4 and 5.5 ​Å. The second constraint was between the terminal carbon of the R36 sidechain (CZ) of ClTx and the sidechain carbonyl carbon of D319 of NRP1-b1, constrained between 5 and 7 ​Å. These distances were based on the NRP1-b1 complex structure with VEGF (4DEQ) (Parker et al., 2012). Finally, a constraint was placed between the terminal nitrogen atom of K27 (NZ) on ClTx and the side-chain carbonyl carbon (CG) of E319 in NRP1-b1, which is found to bind to the first basic residue in the [R/K]-XX-[R/K] motif.

**Fig. 5:**
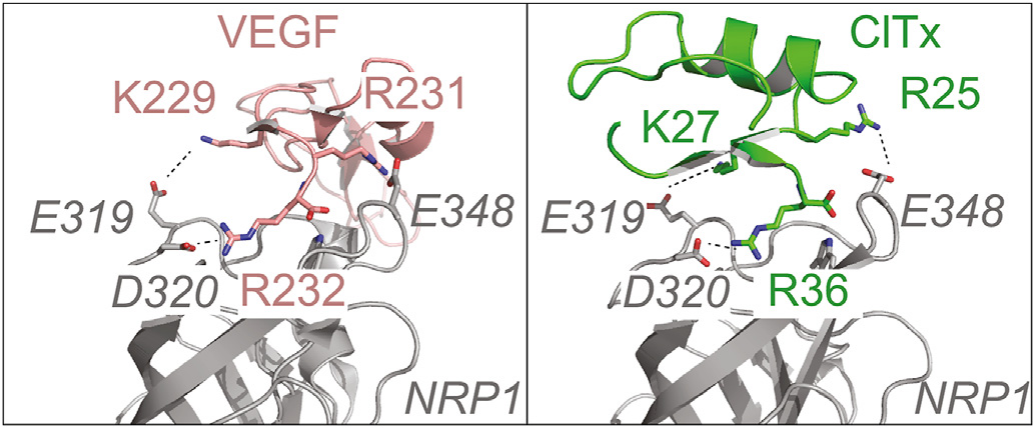
**Endogenous and exogenous ligand binding to NRP1.** Computational docking of the ClTx NMR structure (PDBID: 5L1C) to the NRP1 crystal structure (extracted from PDBID: 5IJR). Distance restraints were generated based on the NMR titration data. The left panel shows the binding of an endogenous ligand (VEGF) to NRP1-b1 via conserved basic residues (K229, R231 and R232). The right panel shows the binding of the exogenous ClTx ligand, based on the NMR data, revealing that residues K27, R25 and R36 occupy positions similar to the corresponding canonical KXRR sequence in VEGF.

The docking produced 7 clusters, and we found that in one of these, the C-terminal arginine overlapped well with the canonical arginine binding pose of NRP1-b1 substrates, while also placing K27 in the vicinity of E319 (see Fig. 5). We were further encouraged that R25 which is perturbed in the NMR data also shows interactions with E348 which can be seen to bind to a basic residue in the [R/K]-XX-[R/K] motif where the penultimate residue is an arginine (as in the case of VEGF). Indeed, we find that this binding pose, overall, explains the observed NMR chemical shift perturbations well.

## 3 Discussion

In this study we developed a new bacterial expression system for production of natively folded, isotope labelled ClTx to study its solution structure and its binding to the vascular endothelial growth factor (VEGF) receptor NRP1. We took advantage of the natural folding machinery of *E. coli* by directing the expressed protein to the periplasm (Klint et al., 2013). Using this system, we were able to recover ~2 ​mg of pure, natively folded peptide, which is similar to the final yield (after refolding) of the previously reported method, which required processing and refolding of inclusion bodies (Wang et al., 2013). Thus, our method presents a more efficient alternative to existing approaches.

To map the NRP1-b1 binding interface of ClTx we solved the high-resolution structure of the peptide using multidimensional NMR methods. The NMR structure overlays well with the recent crystal structure of ClTx (Correnti et al., 2018), while neither compare favourably with the original ClTx structure (1CHL) solved by homonuclear NMR methods (Lippens et al., 1995). In particular, we note that K23 in our structure is consistently proximal to R14, whereas in 1CHL this residue is split between this orientation and in proximity to R25. Similarly, K27 is positioned differently when comparing the two structures, further affecting the surface charge distribution of the peptide (Fig. 2C). The different orientations cause a substantial change in the electrostatic surface of the peptide, where the new structure (5L1C) reveals that residues R14, K15 and K23 form one distinct basic cluster whilst R25, K27 and R36 form a second. In addition to these two patches on the protein surface we note a third, weakly hydrophobic patch, which may be involved in protein-protein interactions spanning residues F6, T7, T8, H10.

The NMR chemical shift mapping experiments reveal a distinct binding interface which includes the basic patch spanning R25, K27 and R36. These results are in qualitative agreement with the report by McGonigle et al., where the binding of ClTx to NRP1-b1 was first reported (McGonigle et al., 2019). However, we find that the ITC binding data using our label free NRP1-b1 to deamidated ClTx shows a notably weaker *K*_d_ (143 ​μM) than measured by biolayer-interferometry of a biotinylated ClTx to NRP1 (1–644) (using an Octet system – *K*_d_ 240 ​nM). This is more likely to reflect inherent differences in the methods used rather than the difference in the materials used.

Modelling of the binding of ClTx to NRP1-b1 suggests that the peptide can spatially satisfy the C-end R rule, via its basic patch. It is, therefore, curious that the binding is relatively weak compared to other substrates (e.g. EG00229, S1 of SARS-CoV-2 and VEGF) (Daly et al., 2020). We note that Y29 and L34 of ClTx are near the interface with NRP1-b1 (in the model and supported by the chemical shift mapping data) without making any favourable interactions. Indeed, it may be that there is some steric hindrance caused by proximity of Y29 to D320 on the receptor. Thus, while the peptide in principle can fulfil the C-end rule, the compact structure of the peptide may introduce a source of unfavorable steric interactions that reduce its binding to NRP1. It, therefore, appears that there may be a possibility to improve the NRP1 binding of ClTx by mutation of residues that are at the binding interface (as revealed by the NMR data).

Further, we note that a number of studies introduce labels on ClTx via conjugation of a reporter to the peptide non-specifically via reaction with free amines on the peptide, which may proceed via reaction with K15, K23, K27 sidechains or the peptide N-terminus (Kievit et al., 2010). While binding to the peptide N-terminus or K15 and K23 are, based on our data, predicted not to affect NRP1 binding, we expect that the modification of K27 may adversely affect this binding. In the study by McGonigle et al., for example, they used a mixture of N-terminal and K27 biotinylated ClTx for NRP1 binding studies (McGonigle et al., 2019). Our model would predict that the two species would show different binding constants. It would appear judicious to either generate a K27R mutant for such studies or generate a more site-specific label that does not interfere with the NRP1 binding interface.

Finally, it is worth considering that NRP1 has been shown to facilitate internalisation of C-end rule cargo (Pang et al., 2014), such as SARS-CoV-2 and VEGF, via micropinocytosis-like pathways, while also leading to transfer of the cargo between cells (Pang et al., 2014). The latter is likely of particular importance for the cancer targeting properties of ClTx. However, considering the known cardiac phenotype of scorpion envenomation (Gueron et al., 1992), we speculate that if ClTx is deamidated in the blood stream it may also be internalised by cardiomyocytes, where NRP1 is known to be present (Wang et al., 2015). Within the cardiac cells, ClTx may ultimately be exposed to the intracellular surface of CLC-3, which is a channel known to be important for cardiac function (Duan, 2011), thus providing a pathway for the peptide to access the receptor that named it, but was dismissed as its natural target (DeBin et al., 1993).

## 4 Materials and methods

### 4.1 Recombinant production of ^13^C/^15^N-labelled ClTx

A synthetic gene encoding ClTx, with codons optimized for expression in *E. coli*, was produced and cloned into the pLIC-MBP expression vector by GeneArt (Regensburg, Germany), resulting in the vector pLIC-ClTx. In this vector, an IPTG-inducible T7 promoter controls expression. The vector further encodes, a maltose-binding protein signal sequence (MalE_SS_) for periplasmic export, a His_6_ tag for nickel affinity purification, a maltose-binding protein (MBP) fusion tag to enhance solubility, and a tobacco etch virus (TEV) protease cleavage site between the MBP and the codon-optimized ClTx gene. Recombinant ClTx was expressed by transforming *E. coli* strain BL21 (DE3) with the pLIC-ClTx plasmid. Cultures were grown in minimal media (MM) containing 47.74 ​mM Na_2_HPO_4_, 22.0 ​mM KH_2_PO_4_, 8.56 ​mM NaCl, 1.6 ​mM MgSO_4_, 80.0 ​nM CaCl_2_, 1.0 ​mg/mL thiamine, 0.2% (v/v) vitamin solution, 50 ​μM FeCl_2_, 2 ​μM H_3_BO_3_, 10 ​μM MnCl_2_, 2 ​μM CoCl_2_, 2 ​μM CuCl_2_, 10 ​μM ZnSO_4_, 2 ​μM Na_2_MoO_4_, 18.7 ​mM ^15^NH_4_Cl, 22.2 ​mM ^13^C_6_-D-glucose and 100 ​μg/mL ampicillin at 37 ​°C with shaking at 250 ​rpm. When the OD_600_ reached ~0.6 the cultures were cooled to 16 ​°C. Protein expression was induced with 100 ​μM isopropyl β-D-1-thiogalactopyranoside (IPTG). Cells were harvested 15 ​h later by centrifugation for 20 ​min at 9110×*g* at 4 ​°C.

For extraction of the His_6_-MBP-ClTx fusion protein from the bacterial periplasm, cells were suspended in TN buffer (40 ​mM Tris, 400 ​mM NaCl, pH 8.0) with a wet cell weight to buffer volume ratio of 1:10, homogenised and sonicated in an ice bath for 15 ​min (counting pulse time only), with 5 ​s pulses intercalated with 8 ​s intervals for cooling. The soluble lysate fraction was isolated by centrifugation at 41,399×*g* for 30 ​min and the fusion protein was captured by passing the soluble lysate over a 5 ​mL prepacked nickel-nitrilotriacetic acid (Ni-NTA) Superflow column (GE healthcare), followed by a wash step with TN buffer containing 30 ​mM imidazole to remove non-specifically bound proteins. The fusion protein was then eluted from the column with 250 ​mM imidazole in TN buffer. The eluate was concentrated by centrifugal filtration in an Amicon Ultra 10 ​K centrifugal filter (Millipore) and pre-equilibrated with TN buffer to remove imidazole. 0.6 ​mM reduced glutathione and 0.4 ​mM oxidized glutathione were added to maintain TEV protease activity while retaining the fold of the peptide (Klint et al., 2013). Approximately 40 ​μg of recombinant His_6_-tagged TEV protease was added per mg of fusion protein, and the cleavage reaction was shaken at room temperature for 18 ​h. The cleaved His_6_-MBP and TEV protease from the cleavage mixture was removed by binding to a prepacked Ni-NTA Superflow resin column (GE healthcare). The initial flow through from the column containing the recombinant ^13^C/^15^N-ClTx was collected, filtered and further purified on a Zorbax 300SB-C3 semi-preparative reversed-phase high performance liquid chromatography (RP-HPLC) column (250 ​× ​9.4 ​mm, particle size 5 ​μm) using a flow rate of 3 ​mL ​min^−1^ and a gradient of 5–80% Solvent B (90% acetonitrile containing 0.043% trifluoroacetic acid (TFA)) in Solvent A (0.05% TFA in water) over 60 ​min. Fractions were collected by monitoring eluent absorption at 214 and 280 ​nm, and lyophilized to obtain pure ClTx (confirmed by MALDI-TOF).

### 4.2 Expression and purification of NRP1-b1 domain

The His_6_-tag construct of human NRP1-b1 domain composed of residues 273 to 586 was expressed and purified using methods described previously (Daly et al., 2020). In brief, the His_6_-tagged NRP1-b1 domain was expressed in Rosetta-gami™ 2 (DE3) cells (Novagen) using Terrific Broth at 37 ​°C followed by ~16 ​h expression at 16 ​°C after IPTG induction. Cell pellets were lysed in lysis buffer containing 50 ​mM Tris-HCl pH 7.5, 300 ​mM NaCl, 50 ​μg/mL benzamidine and DNase I. The purification was carried out using Talon® resin affinity chromatography followed by thrombin cleavage. The His_6_-tag free flow-through fractions were collected and further purified using size-exclusion chromatography (SEC) using a Superdex™ 75 16/60 column (Amersham Biosciences).

### 4.3 MALDI-TOF mass spectrometry

Peptide masses were determined by MALDI-TOF mass spectrometry on a Bruker Autoflex. Fractions obtained from RP-HPLC were mixed (1:1 v/v) with α-cyano-4-hydroxycinnamic acid matrix (5 ​mg/mL in 50% acetonitrile and 1% formic acid) and MALDI-TOF spectra were acquired in positive reflector mode. Masses were reported for monoisotopic [M+H]^+^ ions.

### 4.4 NMR structure determination of ClTx

^13^C/^15^N-labelled recombinant ClTx was dissolved in 20 ​mM phosphate buffer (pH 5.8), 10 ​μM EDTA to a final concentration of 370 ​μM, followed by addition of 5% D_2_O. 10 ​μM DSS was also added to calibrate the chemical shifts. The sample was filtered using a low-protein-binding Ultrafree-MC centrifugal filter (0.22 ​μm pore size; Millipore, MA, USA), and then 300 ​μL was added to a susceptibility-matched 5 ​mm outer diameter microtube (Shigemi Inc., Japan). NMR spectra were acquired at 298 ​K on an NMR spectromter operating at a nominal ^1^H frequency of 900 ​MHz (Bruker BioSpin, Germany) equipped with a cryogenically cooled triple resonance probe.

Sequence specific backbone and sidechain resonance assignments were obtained using 3D CBCA(CO)NH, 3D HNCACB, 3D HNCO, 4D H(CCCO)NH-TOCSY and 2D ^1^H–^15^N HSQC spectra. The 3D and 4D spectra were acquired using non-uniform sampling (NUS) (Mobli and Hoch, 2014). Sampling schedules that approximated the signal decay in each indirect dimension were generated using SCHED3D (Mobli et al., 2010; Mobli, 2015). NUS data were processed using the Rowland NMR Toolkit (www.rowland.org/rnmrtk/toolkit.html) and maximum entropy parameters were automatically selected as previously described (Mobli et al., 2007). ^13^C- and ^15^N-edited NOESY-HSQC experiments were acquired using uniform sampling using a mixing time of 200 ​ms. Separate ^13^C-edited NOESY-HSQC spectra were acquired for the aliphatic and aromatic regions of carbon spectrum.

Backbone dihedral angles (φ and ψ) were derived from the chemical shifts of the backbone atoms using the TALOS-N program (Shen et al., 2009) and the dihedral angle restraint range for the structure calculations was set twice the estimated standard deviation. The disulfide bond connectivities were unambiguously determined based on preliminary structure calculations and included in subsequent structure calculations using distance restraints. NOESY spectra were manually peak picked and integrated, the peak lists were then automatically assigned, distance restraints extracted, and an ensemble of structures calculated using the torsion angle dynamics package CYANA (Güntert, 2004).

### 4.5 ClTx – NRP1-b1 domain interactions and competition with EG00229

#### 4.5.1 *NMR chemical shift mapping*

^15^N-labelled ClTx was dissolved in 50 ​mM sodium citrate (pH 5.5), 150 ​mM NaCl and 5% D_2_O, to a final concentration of 55 ​μM. The ^1^H–^15^N HSQC spectrum of ClTx was acquired at 298 ​K using Bruker Neo spectrometer (900 ​MHz instrument as above). Titration experiments were performed by gradual addition of stock solution (396 ​μM) of NRP1-b1 into ^15^N-labelled ClTx solution, to final NRP1-b1 concentrations of 21 ​μM, 34 ​μM, 45 ​μM, 57 ​μM, 95 ​μM and 125 ​μM.

To map the interaction interface of ClTx and NRP1-b1, competition titrations were performed using a previously characterised NRP1-b1 antagonist – EG00229. To the final titration point of ^15^N-labelled ClTx (55 ​μM) and NRP1-b1 (125 ​μM), concentrated solution (50 ​mM) of EG00229 (dissolved in dimethyl sulfoxide) was added to the final concentration of 83 ​μM. In the subsequent titration, concentration of EG00229 was increased to 413 ​μM.

At each titration point, a ^1^H–^15^N HSQC spectrum was acquired at 298 ​K under identical experimental conditions (using 32 scans). All spectra were processed using Topspin 4.0 (Bruker, Massachusetts, USA) and Rowland NMR toolkit (University of Connecticut, USA). Peak assignments of ^15^N-labelled ClTx were performed using CCPNMR version 3.0.

#### 4.5.2 *ITC measurements*

The interactions of the NRP1-b1 domain with ClTx and EG00229 were performed using a Microcal ITC200 (Malvern) in buffer containing 50 ​mM sodium citrate pH 5.5, 150 ​mM NaCl at 32 ​°C. To test the binding, 800 ​μM of ClTx or EG00229 were titrated into a sample cell containing 40 ​μM of NRP1-b1 domain. Competition assays were carried out by injecting 800 ​μM of ClTx into 40 ​μM of NRP1-b1 domain +200 ​μM of EG00229. Each experiment involved an initial 0.4 ​μL (excluded from data processing) followed by series of 12 injections of 3.22 ​μL each with a stirring speed of 750 ​rpm and a spacing period of 180 ​s between injections. In all binding experiments the equilibrium dissociation constant (*K*_d_), change in binding enthalpy (Δ*H*), change in Gibbs free energy (Δ*G*) and entropy change (Δ*S*) were analysed after fitting data to a single-site binding model with stoichiometry (N) set to 1.0 for calculation.

### 4.6 Modelling of the ClTx – NRP1-b1 domain complex

HADDOCK was used to generate a model of the ClTx-NRP1-b1 complex (Dominguez et al., 2003). The NMR structure ensemble of ClTx (5L1C) was used together with the NRP1-b1 domain from the crystal structure of this protein in complex with VEGF (5IJR). The NRP1-b1 domain was extracted from K115 to T427 of NRP1. The standard HADDOCK parameters were edited to increase the number of structures generated in the rigid body docking from 1000 to 6000 structures, of these 400 were kept (instead of the default 200). The MD simulations steps were increased from 500 to 2000 in the initial high temperature and the first cooling stage of the rigid body docking, and the number of MD steps in the second and third cooling stages were increased from 1000 to 4000. The output was organized in clusters using the HADDOCK analysis scripts based on the RMSD of the docked structures.

## Accession numbers

The structure of ClTx has been deposited to the PDB, accession number 5L1C and the chemical shifts deposited to the BMRB with accession number 30149.

## CRediT authorship contribution statement

**Gagan Sharma:** Conceptualization, Investigation, Resources, Writing – original draft, Writing – review & editing, Visualization. **Carolyne B. Braga:** Conceptualization, Investigation, Resources, Writing – original draft, Writing – review & editing, Visualization. **Kai-En Chen:** Investigation, Resources. **Xinying Jia:** Investigation, Resources. **Venkatraman Ramanujam:** Investigation, Resources. **Brett M. Collins:** Investigation, Resources, Supervision, Funding acquisition. **Roberto Rittner:** Supervision, Funding acquisition. **Mehdi Mobli:** Conceptualization, Investigation, Writing – original draft, Writing – review & editing, Visualization, Supervision, Funding acquisition.

## Declaration of competing interest

The authors declare that they have no known competing financial interests or personal relationships that could have appeared to influence the work reported in this paper.
